# Current methods in explainable artificial intelligence and future prospects for integrative physiology

**DOI:** 10.1007/s00424-025-03067-7

**Published:** 2025-02-25

**Authors:** Bettina Finzel

**Affiliations:** Cognitive Systems, University of Bamberg, Weberei 5, 96047 Bamberg, Germany

**Keywords:** Explainable Artificial Intelligence (XAI), Physiology, Explainability, Interpretability, Survey

## Abstract

Explainable artificial intelligence (XAI) is gaining importance in physiological research, where artificial intelligence is now used as an analytical and predictive tool for many medical research questions. The primary goal of XAI is to make AI models understandable for human decision-makers. This can be achieved in particular through providing inherently interpretable AI methods or by making opaque models and their outputs transparent using post hoc explanations. This review introduces XAI core topics and provides a selective overview of current XAI methods in physiology. It further illustrates solved and discusses open challenges in XAI research using existing practical examples from the medical field. The article gives an outlook on two possible future prospects: (1) using XAI methods to provide trustworthy AI for integrative physiological research and (2) integrating physiological expertise about human explanation into XAI method development for useful and beneficial human-AI partnerships.

## Introduction

Physiology is a diverse field of research within human medicine and beyond. According to Lemoine and Pradeu [57], physiology is an integrative, explanatory science dedicated to overarching phenomena in living beings that are seen as normal (healthy) or pathological (indicative of disease). The British physiologist Noble once pointed out that “physiological analysis requires an understanding of the functional interactions between the key components of cells, organs, and systems, as well as how these interactions change in disease states” [86]. This means physiology is taking a holistic view on living organisms.

Although physiology is a holistic science, it seems that physiological research has not yet exploited the full potential of new technological trends, such as explainable artificial intelligence (XAI) [59], to integrate existing knowledge with new data-intensive analytics. This potential could be utilized in the many research fields that apply physiological methods. This includes research on cardiovascular diseases [33, 49, 84], immunology and homeostasis [102], neural physiology, e.g., for research on Alzheimer’s disease [22] and depression [104], physiology of aging [140] and reproduction [117], cell biology [34] as well as the physiology of nutrition, e.g., in research on human adipogenesis [85], diabetes [141], and effects of diet on cardiovascular diseases [33]. Last but not least, physiology plays a crucial role in tumor detection and diagnosis, e.g., based on histopathological findings using either traditional methods (e.g., TMN-based classification) or modern methods (e.g., immunoscore) [57].

Physiology therefore offers a wide range of applications for artificial intelligence (AI), a set of methods and algorithms including techniques that learn models from data (machine learning), perform automated planning, or provide knowledge representation for automated processing [79, 127]. Most of the AI algorithms currently in use belong to machine learning methods. These can be used for example for regression tasks, classification, or clustering [79, 127]. Their approach is predictive rather than descriptive or functional.

AI is already being used in various areas of physiological research, particularly in oncology, e.g., for the detection and classification of breast cancer [27] and colorectal cancer [95]. AI is also applied for the analysis of cardiovascular conditions, e.g., the detection of systolic dysfunction [29] or in neurophysiology, for tasks like the detection of epileptic seizures [13] and stroke prediction [21].

The vast majority of current AI approaches use so-called deep neural networks (DNN), which offer high-performance architectures but remain opaque to humans in their decisions due to their complexity [87, 126, 131].

Explainable artificial intelligence (XAI), as a field of research and at the same time a term for methods that are either inherently interpretable or represent an interpretable extension of opaque methods, aims to counteract this intransparency [131]. XAI is particularly important for the integration of humans in decision-making processes [87]. XAI methods create understanding in recipients of explanations about the behavior and outcomes produced by AI models, e.g., why a certain cancer type was classified in a given microscopy image [87, 126, 130]. Thus, XAI methods serve experts for the validation of AI models, in particular, to examine whether these models have produced results for valid reasons.

AI models depend on input data and may therefore be prone to noise, errors, or incompleteness in data [87]. In some medical areas, such problems can be amplified by small data sets that cannot be representative of large populations, for example, in histopathology, where false tissue annotations in whole slide images, along with very diverse tissue constellations, can lead to limited generalization in AI models [87]. At the same time, XAI methods can help novices to understand the domain of interest with the help of an AI model, given that it was evaluated as representative by experts.

In physiology and its related medical tasks, human-centered XAI can help to integrate human knowledge into AI models as well as to extract human-understandable concepts from AI models [80, 87, 106]. Established rules (such as TMN cancer classification rules [72]) can be integrated in the process of learning AI models to provide human guidance [126, 130]. Analyzing internal representations of AI models and providing human-understandable labels for them can serve as a first approximation of human knowledge [87, 106]. All in all, putting the human into an explanatory dialogue with AI could lead to improved collaboration and better medical decision outcomes [56, 87].

This review article aims for providing an overview on common XAI topics and methods. It therefore contributes a collection of 85 publications that have been retrieved from a systematic review of 200 papers and articles from the fields of medicine and artificial intelligence with a special focus on physiology. It further illustrates solved and discusses open challenges in XAI research using existing practical examples from the medical field. Based on the extensive review, the article provides a short analysis of the current state of XAI usage in physiology and gives an outlook on two possible future prospects: (1) using XAI methods to provide trustworthy AI for integrative physiological research and (2) integrating physiological expertise about human explanation into XAI method development for useful and beneficial human-AI partnerships. Ultimately, this review article’s goal is to show how XAI could help to meet the integrative demands of physiological research despite the use of data-intensive (opaque) artificial intelligence methods. To the best of my knowledge, this is the first review article that focuses on XAI for physiology and which discusses the potential of XAI to realize integrative physiology.

The review is structured as follows: first, the research area of XAI is introduced with its specific terminology in Section 2. Then, the methodology behind the review of recent publications that utilize XAI in the field of physiology is briefly explained in Section 3. Subsequently, the results of the research are presented and discussed in Section 4. To illustrate common XAI methods and for the discussion of open challenges, examples of XAI methods are shown afterwards, in particular, for medical applications in physiology (see Section 5). The discussion and conclusion Sections (6 and 7) explore and summarize how physiology and XAI as research areas could mutually benefit from each other in the future.

## Explainable artificial intelligence (XAI)

In general, explanatory methods help to create understanding in recipients of explanations (the explainees) about the subject or object that is to be explained (the explanandum) with the help of phenomena or concepts the explanation is based on (the explanans) [57, 126, 131]. In the XAI literature, the component that provides explanations for opaque AI models is often called an XAI method or explainer [131].

According to Carvalho et al. [92] and Schwalbe and Finzel [131], the term explainable artificial intelligence (XAI) has been first used by van Lent et al. in 2004 [59]. The term refers to AI that is capable of explaining itself or that is enhanced by methods that make it understandable to humans. Thus, XAI is the area of research concerned with explaining an AI system’s decision [131]. An idea of this kind has already been coined in 1958 when computer scientist McCarthy proposed inherently transparent AI systems using a formal language and problem-solving algorithms [71, 125].

Early transparent methods led to expert systems research in the 1970s and 1980s until the late 1990s [19], which later lost popularity due to integration challenges. Meanwhile, artificial neural networks became prominent, with efforts to make their decisions transparent beginning in the mid-1990s [31].

The introduction of the GDPR in 2018 emphasized the need for transparency in AI, leading to new frameworks that prioritize accountability and the “right of explanation” [14, 91]. XAI gained further attention after Gunning and Aha’s publication on explainable AI in 2019, which outlined two main goals: creating interpretable models without sacrificing performance and adopting a user-centric approach to enhance human understanding and trust in AI [32].

Significant contributions to the user-centric perspective include Miller’s 2019 paper on social science aspects of AI [78] and Rudin’s advocacy for interpretable models [123]. The XAI community now also focuses on evaluating the quality of explanations, recognizing the importance of formalized assessment metrics [75, 122]. Most recent works provide methods that try to map internal representations in AI models to human-understandable concepts [10, 106, 119] and create explanatory dialogues to allow for multi-faceted interaction and explanation processes [56, 87].

A specific terminology has been established in the XAI community for the categorization of XAI methods [75, 131]. It is briefly introduced in the following list of terms. Note that this review does not aim for providing a comprehensive survey of existing XAI methods. Instead, its goal is to point to current XAI usage in physiological research and to open challenges with respect to integrating explanations with human-centered AI in medical fields, such as physiology.

For a more detailed overview, the interested reader may consider, for example, the recent survey paper by Schwalbe and Finzel (2023) that introduces an XAI taxonomy and presents a collection of over 50 XAI methods [131] and the survey article by Ali et al. [75] that complements XAI topics and methods with their formalization. The following list is an excerpt of terminology adapted from Schwalbe and Finzel [131].

- **Interpretability:** Involves the recognition of constituents of an explanandum and the assignment of meaning to such elements.
- **Explainability:** Refers to the creation of understanding about an explanandum.
- **Model-agnostic explanations:** An explainer produces model-agnostic explanations if it is applicable to any type of model (any type of explanandum).
- **Model-specific explanations:** An explainer produces model-specific explanations if it is applicable to only a certain type of model (a certain type of explanandum). The reason is that such an explainer uses internal model parameters or internal representations to produce explanations.
- **Global explanations:** Provide insights into the reasons for an overall behavior of a model. The explanandum is the model itself. The explanans may be parts of the model or some approximation of the whole model.
- **Local explanations:** Provide insights into the reasons for an outcome of applying a model to input data. The explanandum is the outcome of a model’s application to an individual instance. The explanans may be properties of the input instance (its features) and their influence on the model’s output.
- **Model induction:** This describes the approach of generating a surrogate model based on inputs, constituents, or outputs of another model in order to approximate the explanandum. This term was specifically coined by Gunning and Aha [32].
- **Deep explanation:** This describes the approach of generating explanations based on constituents or outputs of a model, the explanandum, that is usually a very complex and opaque model such as DNNs. This term was coined by Gunning and Aha [32].
- **Interpretable models:** Such models are constructed from interpretable parts and thus usually inherently transparent (see, for example, [32, 123]).
- **Ante hoc explanations:** Explanations are produced during model creation. This applies to interpretable AI models. The model itself can be used to generate global or local explanations.
- **Post hoc explanations:** Explanations are produced after model creation. This applies to opaque AI models which are made transparent with the help of an external explainer. The explainer is used to generate global or local explanations either by accessing internal representations of the model (deep explanation) or by approximating the model’s behavior (surrogate model).
- **Multimodal explanations:** Such explanations are not expressed via a singular modality, e.g., text. Instead, different representations are used (e.g., text in combination with images).
- **Human-centered explanations:** Such explanations address the varying information needs and individual characteristics of human explainees.

This list of terms is not complete as there exists more nuanced terminology for characterizing specialized explainability techniques as well as synonyms that can not be covered altogether in this article. For example, *data explainability* [75] is mentioned as another aspect in the literature to describe methods that enable reasoning on data (e.g., knowledge graphs) or allow the data’s direct characterization (e.g., summarization methods). The term *ante hoc explanations* used in this article is also called *model explainability* (see Ali et al. [75]) in other works. As this article does not discuss explanation evaluation methods, the interested reader may find a comprehensive overview in Schwalbe and Finzel [131] and a concise summary of explanation assessment methods in Ali et al. [75].

In the following sections, the recent works applying XAI to physiological use cases will be collected, partially illustrated, and discussed with respect to their integrative nature. First, the methodology behind the search is shortly introduced.

**Table 1:** Publications (S, survey; M, method) retrieved from query “(explainable artificial intelligence OR XAI OR explainability OR interpretability) AND physiology” with focus (P, physiology; G, general)

#	Reference	Publ. Type	XAI Methods	Published in	Focus
1	Alabdulhafith et al. [97]	M	SHAP, LIME	IEEE Access	P (ICU)
2	Andreu-Perez et al. [143]	M	xMVPA	Commun Biol	P (fNIRS)
3	Anguita-Ruiz et al. [134]	M	Association Rules	PLoS Comput Biol	P (GED)
4	Banerjee et al. [42]	M	SHAP	SN Comp Sci	P (HRV)
5	Beer et al. [26]	M	SHAP	Neurobiol Aging	P (AQP4)
6	Bernard et al. [110]	M	SHAP	Aging Cell	P (PPAge)
7	Boscolo Gal. et al. [139]	S	Various	IEEE Signal Process Mag	P (BrainAge)
8	Boulesteix et al. [96]	M	Regression	Hum Genet	P (SNP)
9	Chan et al. [99]	M	SHAP, PDA, LIME	BMC Med Inform Decis Mak	P (ICU)
10	Chen et al. [54]	M	Integrated Gradients	Cancer Cell	P (WSI)
11	Chen & Chiu [52]	M	Fuzzy Geom. Mean	Patterns	P (Covid)
12	Chen et al. [6]	S	Various	Digital Health	P (NeurImag)
13	Davagdorj et al. [118]	M	DeepSHAP	IEEE Access	P (NCD)
14	Dindorf et al. [93]	M	LIME, SHAP, DeepLift	Sensors	P (Posture)
15	El-Sappagh et al. [137]	M	SHAP	Sci Rep	P (AD)
16	Fellous et al. [16]	S	Various	Front Neurosci	P (NeuroStim)
17	Gao et al. [23]	M	SHAP	Gut Microbes	P (CD)
18	Gimeno et al. [43]	M	MOM	Front Immunol	P (WES)
19	Goodwin et al. [113]	M	SHAP	Nature Neurosci	P (Behavior)
20	Górriz et al. [112]	S	Various	Inf. Fusion	P (Emotion)
21	Gouverneur et al. [3]	M	Grad-CAM	Sensors	P (Pain)
22	Han et al. [89]	M	SHAP, DCA	IEEE ICME Conf.	P (Pain)
23	Hasan et al. [2]	M	SHAP, PDA	Comput Methods Programs Biomed.	P (Driving)
24	He et al. [4]	M	SHAP, PDA	Ecol Ind	P (Seagrass)
25	Hijazi et al. [5]	M	LIME	Sensors	P (Covid)
26	Hossain et al. [11]	S	Various	ACM Comput Surv.	G (Health)
27	Hussain & Jany [12]	M	SHAP, LIME, ACH	Sensors	P (EMG)
28	Islam et al. [18]	M	LIME, Eli5	Sensors	P (EEG)
29	Jaber et al. [20]	M	SHAP	BMC Med Inform Decis Mak	P (Stress)
30	Jiang et al. [25]	M	MDI	IEEE J Biomed Health Inform	P (EMG)
31	Joyce et al. [28]	S	Various	npj Digit. Med.	P (Mental)
32	Juang et al. [30]	M	EFNN	Sleep Med	P (OSAHS)
33	Kalyakulina et al. [36]	S	Various	Ageing Res Rev	P (Age)
34	Keyl et al. [37]	M	LRP, scGeneRAI	Nucleic Acids Res	P (Genes)
35	Khanna et al. [39]	M	Various	Decis Anal J	P (Covid)
36	Khosravi et al. [40]	M	XAI-ED	Comput Educ Artif Intell	P (Biosign.)
37	Kim et al. [41]	M	LRP, SHAP, CAM	Biosensors	P (Biosign.)
38	Kim et al. [44]	M	SHAP	J Med Internet Res	P (CA)
39	Klauschen et al. [45]	S	Various	Annu Rev Pathol	P (Patho)
40	Kumar & Das [46]	M	SHAP	Comput Biol Chem	P (PBMC)
41	Lacalamita et al. [47]	M	SHAP	Int J Mol Sci	P (HCC)
42	Lai et al. [50]	M	SHAP, LIME	Front Immunol	P (AD)
43	Lauritsen et al. [51]	M	DTD, LRP	Nat Commun	P (EWS)
44	Lemańska-P. et al. [55]	M	SHAP, Feature Importance	Cells	P (Sepsis)
45	Li et al. [60]	M	QLattice	npj Microgravity	P (SERCA)
46	Lin et al. [61]	M	SHAP, LIME	Front Med	P (MV)
47	Lisboa et al. [62]	M	EBM	Sci Rep	P (Survival)
48	Liu & Hu [63]	S	Various	Curr Opin Chem Biol	P (RadioGen)
49	Liu et al. [64]	M	SHAP	Lancet Digit Health	P (ICU)
50	Loh et al. [65]	S	Various	Comput Methods Programs Biomed.	G (Health)
51	Lundberg et al. [67]	M	Various	Nature Biomed Eng	P (Blood)
52	Macas et al. [69]	M	Various	Integr Comput-Aided Eng	P (ECG)
53	Madanu et al. [70]	M	Feature Importance	Technologies	P (Pain)
54	Meena & Hasija [73]	M	SHAP	Comput Biol Med	P (Genes)
55	Mei et al. [76]	M	Various	IEEE Trans Evol Comput	G
56	Moulaei et al. [83]	M	SHAP, LIME	Sci Rep	P (Methanol)
57	Novakovsky et al. [88]	M	ExplaiNN	Sci Rep	P (Genes)
58	Novielli et al. [95]	M	SHAP	Front Microbiol	P (CRC)
59	Papadimitroulas et al. [103]	S	Various	Physica Med	G (Onco)
60	Peng et al. [105]	M	SHAP, LIME	J Med Syst	P (Liver)
61	Przepiorka et al. [107]	M	SHAP, LIME	J Neuro-Oncology	P (VS)
62	Qiu et al. [108]	M	SHAP, ENABL Age	Lancet Healthy Longev	P (Age)
63	Ramírez-Mena et al. [111]	M	SHAP	Comput Methods Programs Biomed.	P (PC)
64	Ray et al. [114]	M	Various	Cell Rep Med.	P (Asthma)
65	Roessner et al. [121]	S	Various	Eur Child Adolesc Psychiatry	G (Mental)
66	Rudrapal et al. [124]	M	SHAP, LIME	Mol. Divers.	P (COX-2)
67	Sahoh & Choksuriwong [128]	S	Various	J Ambient Intell Humaniz Comput	G (Health)
68	Sandamal et al. [129]	M	SHAP, LIME	RINENG	P (Fitness)
69	Sganzerla M. et al. [132]	M	SHAP	Sci Rep	P (Proteins)
70	Song et al. [135]	M	SHAP	Nat Commun	P (AKI)
71	Stenwig et al. [136]	M	SHAP	BMC Med Res Methodol	P (ICU)
72	Streich et al. [138]	S	Various	Curr Opin Biotechnol	P (Plants)
73	Talukder et al. [142]	M	Various	Brief Bioinform	P (Genes)
74	Tang et al. [17]	M	Vec2Image	Brief Bioinform	G (Biology)
75	Thorsten-Meyer et al. [1]	M	SHAP	Lancet Digit Health	P (ICU)
76	Tjoa & Guan [15]	S	Various	IEEE Trans. Neural Netw. Learn. Syst.	G (Health)
77	Togo et al. [82]	M	SHAP	J Chem Inf Model	P (Toxicity)
78	Togo et al. [7]	S	Various	Expert Opin Drug Metab Toxicol	P (Toxicity)
79	Veldhuis et al. [109]	M	SHAP, Counterfact	Forensic Sci Int Genet	P (Genes)
80	Wani et al. [35]	M	DeepXplainer	Comput Methods Programs Biomed.	P (LungCancer)
81	Westerlund et al. [120]	S	SHAP, LRP, DTD	Int J Molecular Sci	P (CVD)
82	Wolfe et al. [94]	M	Fuzzy Logic	BMC Genome Biol	P (Genes)
83	Yagin et al. [100]	M	SHAP, LIME	Comput Biol Med	P (Covid)
84	Yang [48]	S	Various	J Healthc Inform Res	G (Health)
85	Zhang [90]	M	LIME	Europ Rev Med Pharmacol Sci	P (Sepsis)

## Review methodology

For this review paper, relevant journals (with impact factor 2.0 or higher) and conference proceedings from the fields of artificial intelligence and medicine with at least an h-index of 10 were searched with the keywords “explainable artificial intelligence, XAI, explainability, interpretability, physiology.” Common platforms (Google Scholar and PubMed) were considered for paper retrieval. Only papers published during the years 2020 to 2024 were included (first considered day, 1.1.2020; last considered day, 1.11.2024). As a further criterion, publications had to have been cited at least *5 times* in the last three years and publications from the past five years at least *10 times* (considering only the year of publication). Preprints and workshop papers were excluded from search results. Papers that referenced applications of XAI in physiology, but did not provide any research outcomes in this area (a survey, method or experimental results), were excluded as well. From the list of results found by PubMed and Google Scholar, only the first 100 entries (sorted by relevance in Google Scholar) have been reviewed. The keywords have been searched with a query using operators. The query was formulated as “(explainable artificial intelligence OR XAI OR explainability OR interpretability) AND physiology” to allow for synonyms and related XAI terms being equally important and to emphasize on physiology.

## Review results for XAI in physiology

All retrieved and included papers and articles have either a strong focus on XAI with mentions of physiology or a strong focus on physiology with applications or substantial discussions of XAI.

Querying the data bases with the aforementioned query generated a list of roughly 24,700 results as denoted by Google Scholar and a list of 51,591 results in PubMed.

From the 100 first entries returned by Google Scholar, 60 met the criteria for the minimum number of citations and for the impact factor of the respective journal or the h-index of the conference. From the 100 first entries returned by PubMed, 53 publications met the citation criterion as well as the minimum impact factor or h-index for journals or conferences. The content was reviewed for its depth in XAI focus or physiology. This left 85 articles, of which 18 were survey papers and 67 presented at least one XAI method.

All selected publications are organized in Table 1. It sorts the alphabetically ordered articles according to (1) their article type being a survey or technical contribution using an XAI method, (2) the XAI method(s) they present^1^, (3) the journal or conference they were published in^2^, and (4) the content focus in medicine (either on physiology or more general with considerable mentions of physiology as application).

Table 1 contains a considerably large number of methodological publications and some survey articles. The majority of the methodological works utilize existing XAI methods, rather than introducing novel ones.

The collection of publications shows neither a prominent research field in terms of journals and conferences, nor a major topic in terms of application areas. The research fields covered in journals and conferences range from multidisciplinary (e.g., IEEE Access), medical (e.g., The Lancet), and microbiological research (e.g., Frontiers in Microbiology) to general, application-oriented research (e.g., Journal of Healthcare Informatics Research).

The publications with a rather broad and general focus and in which physiology plays a subordinate role (denoted by G) cover the topics of health (5 times), mental health (2 times), biology (1 time), and oncology (1 time). The general focus is specified accordingly in Table 1. One of the broad publications (Mei et al. [76]) has a technical focus and was therefore not specified in more detail.

The publications that focus on physiology (P) primarily deal with intensive care medicine (ICU, 5 times), genetics (Genes, 5 times), various forms and measuring instruments of age diagnostics (Age, 4 times), diseases caused by the coronavirus (COVID, 4 times), pain physiology (Pain, 3 times), various biosignals (Biosign., 2 times), Alzheimer’s dementia (AD, 2 times), electromyography (EMG, 2 times), sepsis (2 times), and toxicity (2 times).

Further specialist areas are indicated in the table as corresponding focus topics within physiology. At this point, only those are explicitly mentioned from which the focus—without special prior knowledge—cannot be read directly from the abbreviations used in Table 1. These include functional near-infrared spectroscopy (fNRS), gene expression data (GED), heart rate variability (HRV), the integral transmembrane protein aquaporin-4 associated with amyloid burden in aging brains (AQP4), single nucleotide polymorphism in genetics (SNP), whole slide imaging in (histo-)pathology (WSI), non-communicable diseases prediction (NCD), Crohn’s disease (CD), whole exome sequencing for leukemia (WES), electroencephalography (EEG), obstructive sleep apnea/hypopnea syndrome (OSAHS), cardiac arrest (CA), peripheral blood mononuclear cells as breast cancer biomarkers (PBMC), hepatocellular carcinoma (HCC), early warning scores for critical illness (EWS), cellular calcium homeostasis in muscles through sarcoendoplasmic reticulum calcium ATPase (SERCA), mechanical ventilation in life support (MV), radiogenomics (RadioGen), electrocardiography (ECG), colorectal cancer (CRC), vestibular schwannoma (VS), prostate cancer (PC), bioactivity prediction of phenolic cyclooxygenase-2 inhibitors (COX-2), acute kidney injury (AKI), and cardiovascular disease (CVD).

On closer inspection of the analytical tools utilized in the collected publications, it stands out that some XAI methods were used much more frequently than others. The following numbers were derived for the publications for which the XAI methods are explicitly listed in Table 1. The occurrences in the papers that present a large number of methods simultaneously (mostly surveys) were not counted here, in order to focus more on the application of XAI in physiology. The most frequently used methods include SHapley Additive exPlanations (SHAP) for model-agnostic explanations (40 times) and the Local Interpretable Model-agnostic Explanations (LIME) method (15 times), which are often used in combination. SHAP was mostly used for global explanations and LIME for local explanations. Both methods are model-agnostic and can therefore be widely applied. This probably explains their popularity to a large extent. Layer-wise Relevance Propagation (LRP) as a model-specific explanation method (4 times), Partial Dependency Analysis (PDA) as a model-agnostic explanation method (3 times) as well as Deep Taylor Decomposition (DTD), Class Activation Mapping (CAM, including mostly Grad-CAM and Relevance-CAM in few cases), and feature importance in general (2 times each of the mentioned methods) follow them.

Note that feature importance refers to a wide range of methods that measure and explicate the contribution of features to the final outcome of a computation. There exist different variants, and some of the already mentioned methods, like SHAP, belong to feature importance methods [58], also called attribution methods [131]. The following section briefly introduces the XAI methods that were most common in the reviewed literature.

## Selected XAI methods and examples

Subsequently, the most common XAI methods applied in physiology and collected in Table 1 will be shortly introduced and illustrated. Note that methods that appeared only once in the collected literature, introducing XAI applied to physiological use cases (denoted by P), were omitted in this list. The purpose of the following list is to give a concise introduction to the most important XAI methods *applied* to physiological use cases rather than to give a comprehensive introduction of all kinds of existing XAI techniques. The interested reader may find more information in state-of-the-art survey papers on XAI methods [15, 74, 75, 115, 131].

- **SHapley Additive exPlanation (SHAP)** [66]**:** A model-agnostic game-theoretic method for explaining machine learning model predictions. It assigns importance values, called Shapley values, to input features, quantifying their contribution to a specific prediction relative to a baseline. Based on cooperative game theory, SHAP calculates feature contributions by averaging their marginal contributions across all possible subsets of features. SHAP assumes an additive explanation model where the prediction is expressed as the sum of contributions from each feature and a baseline value. Computing exact Shapley values is computationally expensive; therefore, SHAP employs efficient approximations like KernelSHAP (model-agnostic) and TreeSHAP (optimized for tree-based models). SHAP provides local explanations by attributing contributions to input features for individual predictions, while also offering global insights when aggregated across instances. SHAP can be computationally intensive and sensitive to the choice of baseline. SHAP further requires a definition of features on the input to compute importance values on. It serves as a post hoc explanation method.
- **Local Interpretable Model-agnostic Explanations (LI-ME)** [116]**:** A method for explaining individual predictions of opaque, machine-learned classification models by approximating their behavior locally on input instances with a more simple, interpretable model. LIME works by selecting a specific instance for which the prediction is to be explained, generating a set of randomly perturbed instances around this instance, and using the original model to predict outputs for these perturbations. To focus the explanation on the instance of interest, LIME assigns higher weights to perturbed instances closer to the original. A simple model, such as linear regression, is then trained using weighted features from instances. The interpretable model’s coefficients indicate the importance of each feature in the opaque model’s prediction for the chosen instance. While LIME provides interpretable explanations, they may be valid only in the vicinity of the instance, not generalizing globally. Furthermore, explanations can vary significantly with different perturbation strategies or parameter settings for feature definition. Additionally, its reliance on perturbations and retraining for each instance can make it computationally expensive. Nevertheless, LIME is suitable for use cases, where the ease of interpretation is of great importance. LIME works with different data types (tabular, text, or images). For example, in textual data, the contribution of individual words can be computed, while in images, pixels are grouped into superpixels and randomly switched off (e.g., by replacing them with a neutral background color) to derive the contribution of individual image regions. LIME is in general used for local post hoc explanations.
- **Layer-wise Relevance Propagation (LRP)** [101]**:** A method used to interpret the decisions of DNNs. It works by propagating a model’s prediction backward through the model’s layers to assign relevance scores (importance) to input features (such as individual pixels in an image). These scores represent the contribution of each feature to the network’s output. They can be visualized in the form of *heatmaps* (for images or text). The propagation follows specific rules tailored to the structure and parameters of each layer. The common approach for explanation generation on images is to provide heatmaps based on normalizing and plotting the relevance of every individual pixel. LRP allows to express positive relevance on features (contributing to an output) and negative relevance (speaking against a certain output). A key principle of LRP is the conservation of relevance, which ensures that the sum of relevance scores remains consistent as they propagate from one layer to the next. This principle ensures that the input relevance scores explain the full prediction value. The selection of the aforementioned rules can be non-trivial and may significantly affect the quality and interpretability of the produced explanation. LRP is model-specific, relying on the model’s structure and learned parameters, which makes it less flexible than some model-agnostic methods. Nevertheless, LRP may be more faithful with respect to representing what a model has learned as it has access to model internals. LRP is a local post hoc explanation method. It can be combined with further methods, such as the t-Distributed Stochastic Neighbor Embedding (t-SNE) method introduced by van der Maaten and Hinton [68], to derive more global explanations.
- **Partial Dependency Analysis (PDA)** [38]**:** Computes the effect of a specific input feature on the final output of a model, usually accompanied by a partial dependency plot for visualization [74, 115]. In principle, PDA isolates the relationship between selected features and the model’s output by averaging out the effects of all other features. To perform PDA, one or more features of interest are chosen, and their values are varied across a range. For each combination of values, the model’s predictions are averaged over all possible values of the other features in the dataset. This marginalization removes the influence of irrelevant features. The resulting values are then visualized in a partial dependence plot (PDP), which shows how changes in the selected features affect the model’s predictions. PDA provides global explanations for a model’s behavior [131]. PDA assumes feature independence, which may not hold in cases of highly correlated data, leading to potentially misleading results. Additionally, PDA can be computationally expensive for large datasets or complex models. PDA is a post hoc explanation method.
- **Gradient-weighted Class Activation Mapping (Grad-CAM)** [133]**:** Visualizes for image data which regions of an input image are most important for image classifying convolutional neural network’s (CNN) predictions.^3^ It produces so-called class-specific activation maps, highlighting areas relevant to the model’s decision for a particular class in the form of *heatmaps* (similar to LRP). Grad-CAM first computes a CNN model’s prediction on an input image with respect to the target class. Then, gradients of the predicted target class score with respect to feature maps learned by the CNN are calculated. These gradients represent the contribution of each pixel in the learned feature maps to the class score. To create a class activation map for an input image, weights are computed by averaging the gradients over the spatial dimensions of the feature maps. These weights are further used to produce the class activation map from feature maps. Grad-CAM retains only positive contributions in activation maps (as opposed to LRP). Consequently, the final result is a heatmap that highlights the regions most relevant to the target class. Grad-CAM is class-specific and model-specific. It provides local visual explanations and is a post hoc explanation method for CNNs.
- **Deep Taylor Decomposition (DTD)** [81]**:** Is used to interpret the decisions of DNNs. It is an attribution method that decomposes a network’s output into contributions from the input features. It builds upon *Taylor expansions*, where the neural network’s decision is expressed as a sum of relevance scores assigned to the input features. The idea is to propagate the relevance scores from the output layer back to the input layer, adhering to certain conservation principles. This means that the sum of relevance scores remains consistent across layers, preserving the overall decision value at each stage of the decomposition. Layer-wise Relevance Propagation is built upon the same conservation principle.

**Fig. 1:**
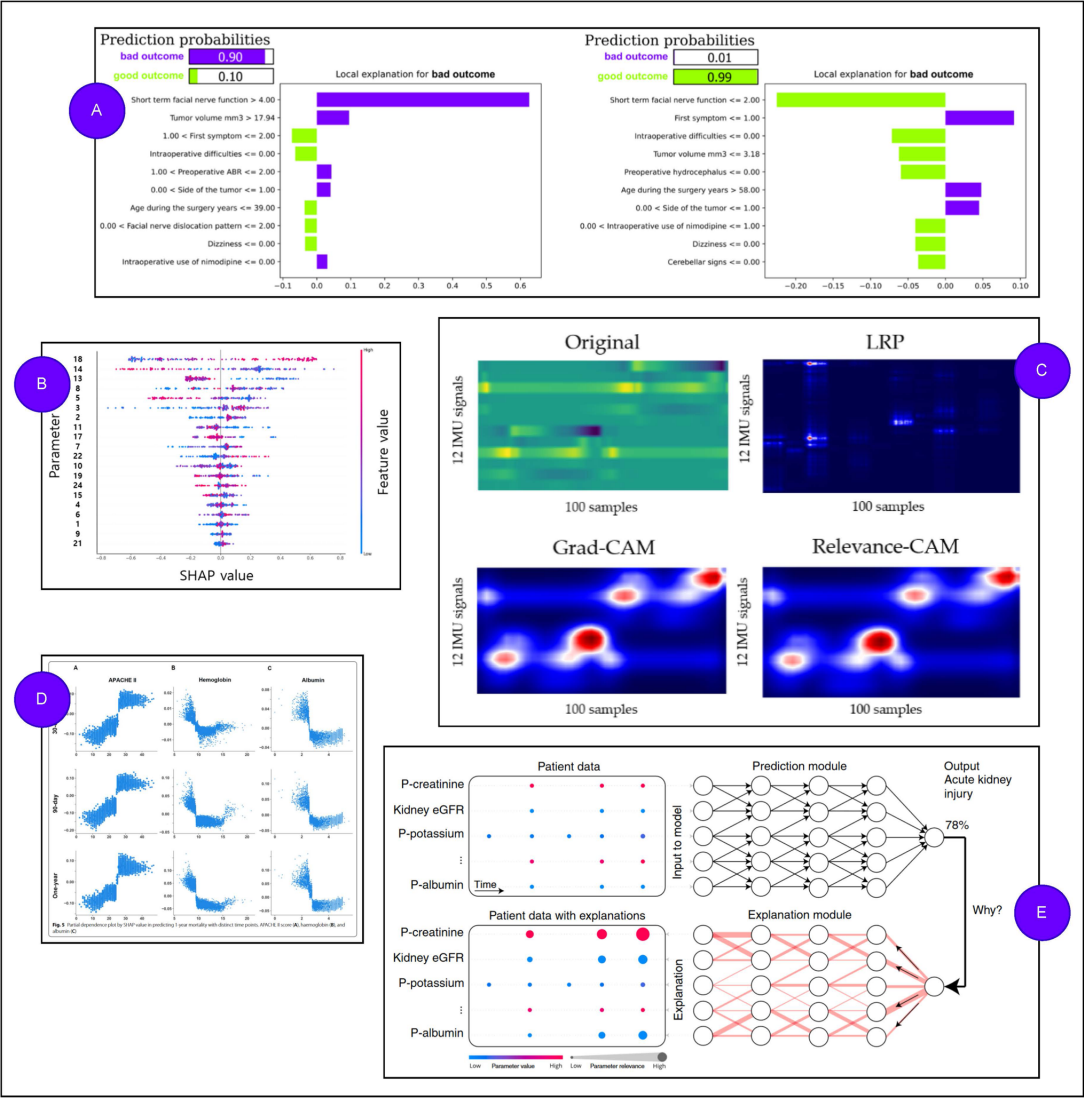
An illustration of visual explanations for AI-based analysis in physiology presenting **A** Local Interpretable Model-agnostic Explanations from Przepiorka et al. [107], **B** SHapley Additive exPlanations from Kim et al. [41], **C** Layer-wise Relevance Propagation, Gradient-weighted Class Activation Mapping and a relevance-based variant from Kim et al. [41], **D** Partial Dependency Analysis from Chan et al. [99], and **E** Deep Taylor Decomposition from Lauritsen et al. [51]

Figure 1 illustrates the explanations produced by the selected XAI methods for some of the works collected in Table 1. All produced explanations are visual, either in the form of a plot or a heatmap. The first displayed visual explanation (A) was produced with LIME for the use case of explaining a classifier for facial nerve functioning after schwannoma surgery as presented in Przepiorka et al. [107]. Another visual explanation (B) was produced with SHAP for sensor-based gait analysis as introduced by Kim et al. [41]. In the same work, Grad-CAM (and Relevance-CAM, a variant of it) and LRP (C) have been used to produce heatmap-based visual explanations. A partial dependency plot (D) was used by Chan et al. [99] to visually explain the mortality prediction of a machine-learned model with the help of PDA. A visual explanation produced by Deep Taylor Decomposition (E) was presented by Lauritsen et al. [51] for the prediction of acute critical illness from different early warning scores.

After having presented the most important works on XAI in physiology and having illustrated selected methods applied to physiological use cases, the next section discusses which XAI-specific challenges in physiology still remain open from an XAI point of view and what implications the respective findings have for the existing methods and their further development.

## Discussion

In the introduction, it was motivated that in addition to existing XAI methods for physiology, open challenges in the further development of XAI for physiology and the improvement of XAI through physiological knowledge should be considered.

One finding is that the majority of the methodological works that have been reviewed utilize existing XAI methods, rather than introducing novel ones. This may suggest that the focus of medical research is currently more on solving a domain-specific problem and justifying the solution with existing XAI methods rather than presenting XAI as a novel solution to physiological problems.

According to Lemoine and Pradeu (2018), clinical phenomena (considered the explanandum) may be first explained by physiological phenomena (which are the explanans here). These physiological phenomena (now considered the explanandum) can then be explained by molecular phenomena (explanans) or by phenomena described by other sciences [57]. Physiology and related fields could therefore benefit from combining XAI methods in an integrative manner to derive new conclusions or achieve the representation of more complex knowledge with the help of explainability.

Considering the examples presented in Section 5, a major finding of this work is that many visual explanation methods are applied or developed for physiological use cases. There seems to be a lack of multimodal approaches that combine visualizations and, for example, verbal statements for more expressive explainability. Multimodal explanations [24] as well as interactive explanations, such as bi-directional explanatory dialogues [56, 126, 130], are considered important prerequisites to human-centered explanations and could be implemented with interpretable models that provide ante hoc explanations [87].

The current XAI methods applied to physiology seem to utilize only static explanations rather than interactive ones. Human-centered approaches that integrate the human as an active participant into AI-supported decision-making processes and that allow for introducing human expertise into learned models could be addressed in future development attempts [87]. Another advantage could be that *human-in-the-loop* systems may drive faster implementation of AI-supported analysis [8, 98].

Furthermore, Lemoine and Pradeu (2018) argue that data-driven prediction methods do not seek explanations in the way traditional physiology does. They state that prediction-based approaches in general may “inspire, and be inspired by, physiology, but are not themselves physiological, in that they do not focus on explanation” [57].

XAI could build a bridge by revealing concepts in features underlying the outcome (prediction) of a data-intensive model. These concepts could be the building blocks that form the basis of constructing more complex explanations [10].

As stated by Lemoine and Pradeu in this context, “Clarity can only be achieved by placing knowledge gathered in non-physiological approaches (remark: e.g., statistical, data-driven models) into a framework, by integrating it into a physiological picture” to derive new explanations and knowledge bases [57].

Integrating results and findings produced with the help of XAI is thus not only the way to follow for evaluating data-intensive models against complex knowledge domains like physiology, but XAI could also provide the means for future knowledge discovery in physiology with the help of AI.

It is furthermore worth mentioning that generative artificial intelligence (genAI) has the potential to break new diagnostic ground and make outcomes transparent with the help of XAI [77]. The work considered in this paper is based on more traditional AI and XAI methods, well established and justified for the respective domains. Under consideration of the advancements in regulatory developments, it is expected that there will be an increase in the use of genAI in medicine over the next few years [77].

Finally, the explanation itself is a physiological process with neural and biochemical events occurring in memory, attention, and perception of human cognition. XAI research could benefit from considering neurological constraints and potentials in reasoning about and production of explanations in humans [9].

## Conclusion

This article reviewed recent publications using explainable artificial intelligence (XAI) in physiology. It provided a collection of 85 works, introduced and illustrated the most applied XAI methods, and discussed open challenges regarding the interfaces between XAI and physiology. Specifically, physiology could benefit if XAI methods were used to conduct knowledge discovery and to integrate human knowledge with models and data. XAI could benefit from considering physiological knowledge for improving generated explanations. Ultimately, providing human-centered explanations for AI decisions could pave the way to a more integrative usage of AI in physiology.

## Data Availability

No datasets were generated or analyzed during the current study.

## Footnotes

1 Note that for survey articles, the full list is omitted and instead denoted with *various*.

2 Note that long journal or conference names are presented in abbreviated form for space reasons.

3 The principle of convolutional neural networks was introduced by LeCun et al. [53].

## Code availability

For some of the referenced works, code is available.
